# Protocol for quantification of AAV-CRISPR-mediated hepatic gene editing in mice

**DOI:** 10.1016/j.xpro.2026.104849

**Published:** 2026-09-29

**Authors:** Kelsey E. Jarrett, Rodrigo Baltazar-Nunez, Chloe Anne Borja, Precious Juvie Calderon, Rochelle W. Lai, Angela Cheng, Manasvi Paudel, Gabriella E. Rubert, Anthony Suberski, Owen K. Traina, Sunny Y. Cai, Kalyani Srikanth, William R. Lagor, Elizabeth J. Tarling, Thomas Q. de Aguiar Vallim

**Affiliations:** 1 Division of Cardiology, Department of Medicine, University of California, Los Angeles, Los Angeles, CA 90095, USA; 2 Department of Biological Chemistry, David Geffen School of Medicine, University of California, Los Angeles, Los Angeles, CA 90095, USA; 3 Department of Integrative Physiology, Baylor College of Medicine, Houston, TX 77030, USA; 4 Jonsson Comprehensive Cancer Center (JCCC), University of California, Los Angeles, Los Angeles, CA 90095, USA; 5 Molecular Biology Institute, University of California, Los Angeles, Los Angeles, CA 90095, USA

**Keywords:** Metabolism, Molecular Biology, CRISPR

## Abstract

Adeno associated viral (AAV) delivery of CRISPR-Cas9 enables efficient, targeted hepatic gene disruption composed of both small insertions and deletions (INDELs) and larger DNA structural variants (DNA-SVs), which are difficult to quantify. Here, we present a protocol for quantifying AAV-CRISPR-mediated gene editing in mice. We describe guide RNA (gRNA) design, plasmid cloning, delivery of AAV-CRISPR to the liver, and execution of liver DNA-editing analysis. This protocol enables quantification of INDELs and DNA-SVs, allowing for validation of gene disruption.

For complete details on the use and execution of this protocol, please refer to Chan et al.^1^

## Highlights

• Protocol for quantifying AAV-CRISPR mediated gene editing in murine liver
• Instructions for designing gRNAs and delivering AAV-CRISPR to the liver
• Procedures for indirectly quantifying large DNA structural variants by qPCR
• Combined DNA-SV qPCR and sequencing enable comprehensive quantification of gene editing

## Abstract (editor highlights)

Publisher’s note: Undertaking any experimental protocol requires adherence to local institutional guidelines for laboratory safety and ethics.

## Graphical abstract

**Figure:**
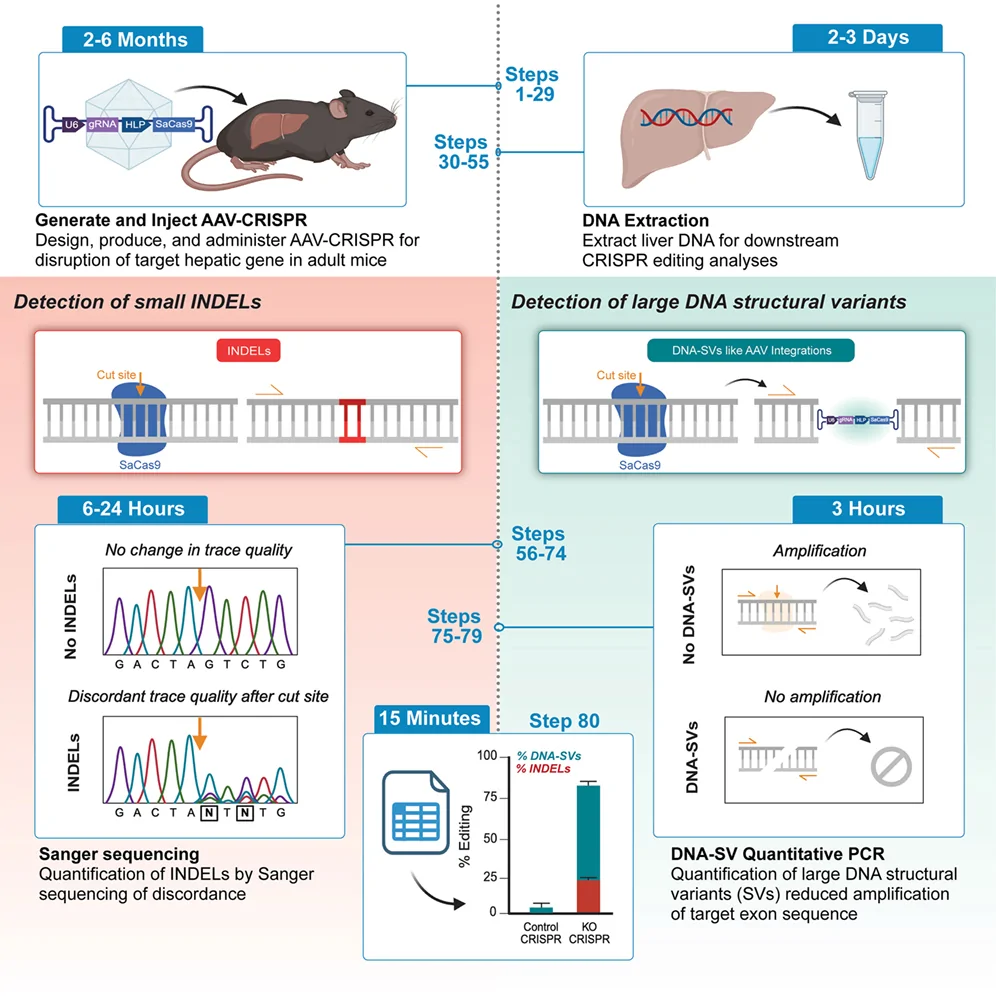

## Before you begin

The protocol below describes the steps used to design, produce, and validate adeno associated virus (AAV) delivery of clustered regularly interspaced short palindromic repeats Cas9 (CRISPR/Cas9) tools for hepatic disruption of genes in adult C57BL/6J mice. In our recent study,^1^ we successfully used this approach to target genes involved in bile acid synthesis, *Cyp7a1, Cyp8b1, Cyp2c70,* and *Cyp2a12*. This protocol provides detailed instructions for CRISPR guide RNA (gRNA) design and validation for *Staphylococcus aureus*-derived Cas9 systems.

Current methods to assess AAV-CRISPR-mediated editing in the liver primarily quantify small, 2–3 bp DNA insertions and deletions (INDELs) by Sanger sequencing or next generation sequencing (NGS) measurements of discordance.^2^^,^^3^ However, these approaches consistently underestimate total editing efficiency in the liver. In our studies, maximal editing efficiency by these methods is typically less than 50%, despite undetectable protein levels by western blotting or ELISA. Given that hepatocytes comprise 70%–80% of the liver,^4^^,^^5^ this level of editing is lower than expected, suggesting that a substantial fraction of editing events are not captured by conventional sequencing-based approaches.

We and others have documented integrations of the inverted terminal repeat (ITR) from the AAV-CRISPR genome into the CRISPR cut site.^6^^,^^7^^,^^8^^,^^9^^,^^10^^,^^11^^,^^12^^,^^13^ While small ITR integrations can be captured through NGS methods, whole AAV integrations may account for up to 47% of editing events.^14^ These integrations include partial or entire AAV genomes, over four kilobases, which are too large to be effectively captured by these NGS-based methods or standard polymerase chain reaction (PCR) for direct quantification. In addition, though AAV integration is thought to be the dominant modification when AAV is used for delivery, large deletions and translocations are known to occur during repair of CRISPR/Cas9-mediated double-stranded DNA cutting. These CRISPR-mediated DNA structural variants (DNA-SVs) have been challenging to quantify.

Here, we describe a technique for indirectly quantifying these DNA-SV events, allowing for comprehensive quantification of CRISPR-mediated editing in combination with Sanger sequencing-based analysis of small INDELs. We developed a quantitative PCR (qPCR)-based method that measures amplification efficiency of the gRNA target locus at the DNA level. We amplify a 400 bp fragment surrounding the gRNA target site, where large DNA-SV modifications like integration of the AAV genome (up to 4.6 kb) cannot be amplified effectively during short qPCR cycles. Thus, if disruption by CRISPR occurs results in DNA-SV events, qPCR amplification efficiency is decreased due to these modifications. This difference in amplification allows indirect prediction of the percentage of DNA-SV events. We then complete small INDEL quantification using Sanger sequencing with the same primer set. The combination of AAV integration with INDELs provides a quantitative measure of editing events that can approach 70%, suggesting most hepatocytes have been edited. Together, our approaches quantify both of the most common AAV-CRISPR-mediated hepatic genome editing events together for the first time.

### Innovation

Quantification of CRISPR-mediated editing at specific target sites has previously been incomplete, as it excluded measurements of CRISPR-mediated DNA structural variants that include AAV integrations. Existing approaches relying solely on Sanger or next-generation sequencing (NGS) cannot effectively capture these large DNA modification events at the CRISPR cut site. This leads to underestimation of total editing and incomplete assessment of the true efficacy of specific gRNAs for successful CRISPR targeting. Here, we incorporate a simple qPCR-based method to detect and quantify DNA-SV events alongside conventional INDEL detection, providing a quantitative analysis of editing outcomes at the DNA level.

This workflow consists of three key components: (1) optimized CRISPR gRNA design, (2) *in vivo* testing for gRNA validation, and (3) quantification of both DNA-SV events and INDEL events at the DNA level. This comprehensive approach enables improved gRNA design and generates comprehensive quantification of AAV-CRISPR editing efficiency. Together, the combination of the techniques in our protocol can inform decision-making on the effectiveness of using hepatic AAV-CRISPR to target genes of interest.

### Institutional permissions

The procedures in this protocol were performed in accordance with the Institutional Animal Care and Use Committee (IACUC) and approved by the Animal Research Committee at the University of California Los Angeles. For completion of these research activities, researchers need to obtain the necessary approvals from the relevant institutional committee at their home institution prior to commencing this research protocol.

## Key resources table

**Table:** 

REAGENT or RESOURCE	SOURCE	IDENTIFIER
**Bacterial and virus strains**		

NEB Stable Competent *Escherichia coli*	New England Biolabs	Cat #C3040H
AAV serotype 2/8 (expressing AAV-CRISPR)	This paper	N/A

**Chemicals, peptides, and recombinant proteins**		

BbsI-HF restriction enzyme	New England Biolabs	Cat#R3539S
XmaI restriction enzyme	New England Biolabs	Cat#R0180S
SnaBI-HF restriction enzyme	New England Biolabs	Cat#R0130S
PvuII-HF restriction enzyme	New England Biolabs	Cat#R3151S
CutSmart Buffer (10×)	New England Biolabs	Cat#B6004S
T4 DNA Ligase	New England Biolabs	Cat#M0202S
T4 DNA Ligase Buffer (10×)	New England Biolabs	Cat#B0202S
KAPA HiFi PCR Kit (or similar)	Roche/KAPA Biosystems	Cat#KK2502
2× SYBR qPCR Master Mix (e.g., Roche LightCycler 480 SYBR Green I Master or AzureBlue 2× qPCR Mix)	Roche or Azure Biosystems	N/A
Proteinase K (10 mg/mL)	Qiagen (or equivalent)	Cat#19133
Phosphate-buffered saline (PBS), sterile	Any supplier	N/A
Dimethyl sulfoxide (DMSO)	Any supplier	N/A
Ethidium bromide or equivalent DNA stain	Any supplier	N/A
Cesium chloride (for AAV purification, molecular biology grade)	Any supplier	N/A

**Critical commercial assays**		

DNeasy Blood and Tissue Kit	Qiagen	Cat#69504
QIAquick Gel Extraction Kit	Qiagen	Cat#28704
QIAprep Spin Miniprep Kit	Qiagen	Cat#27104
HiSpeed Plasmid Maxi Kit	Qiagen	Cat#12662
RNA Extraction Kit (e.g., Qiagen RNeasy Mini Kit)	Qiagen or equivalent	Cat#74104
cDNA Synthesis kit with random hexamer priming	Any Supplier	N/A

**Experimental models: Organisms/strains**		

Mouse: C57BL/6J (males or females, 8 weeks of age)	The Jackson Laboratory	JAX#000664; RRID: ISMR_JWX:000664

**Experimental models: Cell lines**		

HEK293T (for AAV production)	ATCC	Cat#CRL-3216; RRID: CVCL_0063

**Oligonucleotides**		

*Cyp7a1* gRNA6 top oligo: 5′-CACC gtcctaaatcaaagagcgctgt-3′	This Paper	N/A
*Cyp7a1* gRNA6 bottom oligo: 5′-AAAC acagcgctctttgatttaggac-3′	This Paper	N/A
*Cyp2c70* gRNA4 top oligo: 5′-CACC ggctggttctactgagtaacg-3′	This Paper	N/A
*Cyp2c70* gRNA4 bottom oligo: 5′-AAAC cgttactcagtagaaccagcc-3′	This Paper	N/A
*Cyp2c70* gRNA5 top oligo: 5′-CACC gctaagccttggctggttcta-3′	This Paper	N/A
*Cyp2c70* gRNA5 bottom oligo: 5′-AAAC tagaaccagccaaggcttagc-3′	This Paper	N/A
SaCas9 titering Forward: CCGTCGTGAAGAGAAGCTTCATC	De Giorgi et al.^15^	N/A
SaCas9 titering Reverse: CCACCTCATAGTTGAAGGGGTTG	De Giorgi et al.^15^	N/A
Jax 31704 Internal Positive Control Forward: AGTGGCCTCTTCCAGAAATG	Jackson Laboratories	N/A
Jax 31705 Internal Positive Control Reverse: TGCGACTGTGTCTGATTTCC	Jackson Laboratories	N/A
U6 sequencing primer: CCTTCATATTTGCAT ATACGATACAAGGCTGTTAG	De Giorgi et al.^15^	N/A
*Cyp7a1* Genomic Editing Region Forward (KEJ_0140): 5′-CACTAGCTAGCCCGAGTTTAGC-3′	This Paper	N/A
*Cyp7a1* Genomic Editing Region Reverse (KEJ_0141): 5′-CAAGTGAATAGGGACGCCCGC-3′	This Paper	N/A
*Cyp2c70* Genomic Editing Region Forward (KEJ_0152): 5′-GGTGAGGGAACAACCTCACAG-3′	This Paper	N/A
*Cyp2c70* Genomic Editing Region Reverse (KEJ_0153): 5′-CAGTGACAAGAGCGAAGGCTG-3′	This Paper	N/A
*Cyp7a1* mRNA Editing Region Forward (KEJ_0543): 5′-TTCTGCGAAGGCATTTGGAC-3′	This Paper	N/A
*Cyp7a1* mRNA Editing Region Reverse (KEJ_0544): 5′-CTTTCCCGGGCTTTATGTGC-3′	This Paper	N/A

**Recombinant DNA**		

AAV destination plasmid with U6 promoter and BbsI cloning site (e.g., 1313_pAAV-U6-SA-BbsI-MluI-gRNA-HLP-SACas9-HA-OLLAS-spA)	Addgene	Cat#109314; RRID:Addgene_109134
pAdDeltaF6 (AAV helper plasmid)	Addgene	Cat#112867; RRID:Addgene_112867
pAAV2/8 (AAV serotype 2/8 packaging plasmid)	Addgene	Cat#112864; RRID:Addgene_112864

**Software and algorithms**		

CRISPOR (gRNA design)	Concordet & Haeussler, 2018^16^	https://crispor.gi.ucsc.edu/RRID:SCR_015935
NCBI Primer-BLAST (primer specificity)	NCBI	https://www.ncbi.nlm.nih.gov/tools/primer-blast/
UCSC Genome Browser	UCSC	https://genome.ucsc.edu/RRID:SCR_005780
SnapGene or Benchling (DNA annotation)	SnapGene/Benchling	https://www.snapgene.com/RRID_SCR_01502
EditCo ICE Analysis (INDEL quantification)	EditCo Bio^3^	https://www.editco.bio/crispr-analysis RRID:SCR_024508
Graphpad Prism V10	Graphpad	https://www.graphpad.com/
Excel	Microsoft	N/A

**Other**		

NanoDrop spectrophotometer	Thermo Fisher Scientific	N/A
Roche LightCycler 480 qPCR instrument (or equivalent)	Roche	Cat#05015243001
384-well qPCR plate with optically clear sealing tape	Any supplier	N/A
Bead homogenizer (e.g., Omni Bead Ruptor)	Omni International or equivalent	cat#19-042E
Thermocycler	Any supplier	N/A
Gel electrophoresis system with UV transilluminator	Any supplier	N/A
Microcentrifuge (capable of 10,000 × g)	Any supplier	N/A

## Materials and equipment

Obtain a sufficient number of mice for AAV-CRISPR testing. n=3–6 mice/group were used for gRNA validation prior to the studies in Chan et al.^1^

Prepare equipment, supplies, and chemicals for designing and cloning AAV-CRISPR plasmids, DNA analysis, and optional RNA analysis.

• Computer with internet access (for NCBI, UCSC Genome Browser, and CRISPOR).
• DNA annotation software like SnapGene or Benchling.
• Access to NCBI or UCSC Genome Browser databases.
• NEB restriction enzymes: BbsI-HF, XmaI-HF, SnaBI-HF, and PvuII-HF.
• NEB 10× CutSmart Buffer.
• AAV destination plasmid containing U6 promoter and BbsI flanked cloning site (e.g., Addgene #1093144).
• DNA oligonucleotides for gRNA cloning (“Top” and “Bottom” strands with CACC/CAAA overhangs).
• Nuclease-free water.
• 1.5 mL microcentrifuge tubes.
• 10× Annealing Buffer.

**Table:** 

Stock reagent	Amount	Final concentration
1M Tris-HCl, pH 7.5	5 mL	100 mM Tris-HCl pH 7.5
5M NaCl	10 mL	1M NaCl
0.5 M EDTA (pH 8)	1 mL	10 mM EDTA
Water	34 mL	–
**Total**	50 mL	–

Store at 25°C.

• Boiling water bath (at least 95°C).
• Foam tube floats (for annealing step).
• Agarose.
• PCR strip tubes (0.2 mL).
• Gel electrophoresis tank and power supply.
• DNA loading dye.
• Scalpel or razor blade for excising gel bands.
• Ultraviolet light wand or gel box.
• DNA Gel Extraction Kit (e.g., Qiagen Gel Extraction Kit).
• NanoDrop Spectrophotometer for DNA quantification.
• NEB T4 DNA Ligase and 10× T4 Ligase Buffer.
• NEB Stable Competent *Escherichia coli.*
• Luria Broth (LB, antibiotic-free).
• LB + Ampicillin plates (100 μg/mL).
• Shaking incubator (37°C).
• Miniprep and Maxiprep kits for plasmid DNA isolation.
• DNA isolation Kit (e.g., Qiagen DNeasy Blood and Tissue Kit).
• Multichannel repeater pipette.
• 384 well assay plate for quantitative PCR.
• Optically clear tape.
• Primers specific to the control genomic region and the region of editing.
• 2× SYBR master mix.
• High fidelity polymerase PCR kit (e.g., Kapa Hi-Fi PCR Kit).
• Dimethyl sulfoxide (DMSO).
• RNA Extraction Kit (e.g., Qiagen RNA Blood and Tissue Kit).

## Step-by-step method details

### Guide RNA design and cloning of AAV-CRISPR plasmids

**Timing: 2–3 weeks**

This section covers annotation of DNA sequences for gRNA selection, gRNA design, and cloning into the AAV-CRISPR vector. Plasmid annotations completed here are important at later steps for primer design and for editing analysis. The gRNAs are placed in the first half of the coding sequence of the gene of interest, using a combination of manual annotation and online tools to ensure specificity and compatibility with the AAV-CRISPR system for gRNA expression. Finally the gRNAs are ordered as oligonucleotides with overhangs complementary to restriction sites in the AAV-CRISPR cloning vector. Cloning is performed using standard sticky-end cloning with T4 DNA ligase, and the completed plasmids can then be used for AAV production.

1. Download and annotate the mRNA and genomic files for the gene of interest (Figure 1) from NCBI or UCSC Genome Browser. For simplicity, here are the instructions for NCBI:
  a. Download known mRNA variant files and the genomic file as Genbank format to retain annotations for use in SnapGene or other software.
  b. Align mRNA files and identify early coding exons in the first half of the gene that are common across expressed isoforms in the liver.
    ***Note:*** For smaller genes with one or two exons, the whole coding region should be considered for CRISPR gRNA design with special emphasis on essential domains.
  c. Annotate individual exons in the genomic DNA file and define exons within the coding region.
  d. Annotate repetitive or conserved regions, like signal peptides, that will be common across many proteins. These regions should be avoided for gRNA design.
2. Design gRNAs specific to the gene of interest using software that can predict specificity.
  ***Note:*** We have previously covered these steps,^15^ but provide an updated protocol here. The design pipeline uses CRISPOR,^16^ but many of these steps can be applied to other design suites.
  **CRITICAL:** Design for the smaller *Staphylococcus aureus* Cas9 uses different predictors and a different protospacer adjacent motif (PAM), NNGRRT, than the widely used (and larger) *Streptococcus pyogenes* Cas9, which commonly uses a NGG PAM. Ensure that the parameters for the correct gene editor are utilized for gRNA design.
  a. Identify coding exons within the first half of the coding region that contain PAM motifs for SaCas9, NNGRRT.
  b. Copy and paste a candidate exon into CRISPOR https://crispor.gi.ucsc.edu/ and execute the search:
    i. Label the gene name and exon number on the search page for easier identification later.
    ii. Select the correct genome (e.g., Mus Musculus UCSC.2011).
    iii. Select the correct PAM: 21 basepair (bp)-NGG(A/G) (A/G)T – Cas9 *S. aureus.*
      ***Note:*** This protocol centers around a 21 bp gRNA length, but it should be effective for other gRNA conformations where the CRISPR disrupts double-stranded DNA. Select the appropriate CRISPR system for the required application.
      ***Note:*** Other SaCas9 variants exist which have relaxed NNNRRT^17^ or NNG^18^ PAMs. They can be designed in similar fashion, but with alternate constraints for gRNA selection.
  c. CRISPOR will generate a list of potential gRNAs. Consider gRNAs that the program highlights in green with the following score parameters:
    i. Specificity Score: 75+.
    ii. Efficiency Score: 75+.
    iii. Out of Frame: 50+.
      ***Note:*** A high specificity score suggests fewer off-target events while a high efficiency score reflects gene disruption is likely to occur rapidly at that gRNA site. The out of frame score increases confidence that the targeted disruption will shift the reading frame to generate a premature stop codon.
      ***Note:*** Since 2024, the efficiency score in CRISPOR is unavailable for SaCas9. This information is left here in case it returns in a future CRISPOR update. In general, a high specificity score is sufficient to select effective gRNAs.
  d. Further narrow selected gRNA list:
    i. CRISPOR performs *in silico* off target analysis. Exclude gRNAs with off-target sites in exons or without mismatches near the PAM.
    ii. Exclude gRNAs labeled as, “Not with U6/U3” as those gRNAs have quadruple T repeats which terminate transcription from U6.
    iii. Avoid repeats of three or more of the same nucleotide within ten bases of the protospacer adjacent motif.
      **CRITICAL:** For the vector Addgene #109314,^1^^,^^8^ a 5′ G is not included downstream of the U6 promoter. To ensure successful expression of the gRNA from the U6 promoter, if a G is not present in the gRNA sequence, one should be added to the 5′ end (furthest from the PAM), generating a 22 bp gRNA.
      ***Note:*** Selecting at least one high-scoring gRNA with a natural 5′ G is advisable in case adding the G negatively impacts gRNA processing.
3. Annotate 3–5 selected gRNAs in the genomic and mRNA sequence files, ensuring that gRNAs have been selected in more than one exon when possible. Label each gRNA and PAM to clearly indicate whether the gRNA is on the top or bottom strand (Figure 1).

***Note:*** The selected gRNAs may not be the top scoring gRNAs after considering off targets and other features in the gRNA list.

***Note:*** Annotating gRNA location in the genomic and mRNA files will facilitate amplicon design for DNA and RNA analysis in Steps 18–22.

4. Generate plasmid files containing the gRNA (Figure 2).
  a. In the plasmid file for the vector the gRNA will be cloned into (Figure 2A), identify the BbsI restriction sites. The gRNA will be placed in between the cut sites as shown in Figure 2B.
    **CRITICAL:** The vector used here (Addgene #109314^1^^,^^8^) uses BbsI for gRNA cloning. Other vectors use different restriction sites, such as BsmbI or BsaI.
  b. Orient the gRNA from 5′ to 3′ so that the PAM would be 3′ from the gRNA, but do not insert the PAM into the plasmid file.
    **CRITICAL:** If the gRNA does not contain a 5′ G, one should be added in the plasmid file at this step.
  c. If the gRNA originally exists on the bottom strand of the DNA, the sequence must be inserted as reverse complement in the 5′–3′ direction.
5. Order oligonucleotides for gRNA cloning for the top 3 gRNAs.
  ***Note:*** Oligonucleotides can be ordered from any commercial company, such as Integrated DNA Technologies or Sigma Aldrich.
  a. Order the “Top” oligonucleotide with a 5′ CACC overhang: 5′**CACC**NNNNNNNNNNNNNNNNNNNNN-3′
  b. Order the “Bottom” oligonucleotide with a 5′ AAAC overhang. This is the reverse complement of the gRNA plus CAAA: 5′**AAAC**NNNNNNNNNNNNNNNNNNNNN-3′
    **CRITICAL:** If the addition of a 5′ G is necessary, the overhang for the Top Oligonucleotide should be 5′-CACCG-gRNA-3′ leading up to the gRNA, and the Bottom Oligonucleotide 5′-gRNA-GCAAA-3′
    **CRITICAL:** Use the correct overhangs to generate the sticky ends if a different cloning site is used. The bases may not be CACC or CAAA for other plasmids.
    ***Note:*** By making the plasmid file first, the Top and Bottom oligonucleotides can simply be copied from the plasmid file to prevent mistakes transcribing the sequence. The Bottom oligonucleotide must be copied as reverse complement from 5′ to 3’.
6. Linearize the destination plasmid with BbsI-HF to prepare the cut vector for cloning.
  a. Set up the 50 μL digest mix:

**Table:** 

Reagent	Amount
AAV Destination Plasmid	6–8 μg
10× NEB Cutsmart Buffer	5 μL
Water	Adjust total volume to 48 μL
BbsI-HF (NEB)	2 μL
  b. Perform the restriction digest using a thermocycler:

**Table:** 

Steps	Temperature	Time
Incubation	37°C	2–4 h
Heat inactivation	65°C	15 min
Hold	4°C	indefinitely
    **CRITICAL:** Do not use Alkaline Phosphatase to dephosphorylate the cut sites on the plasmid. The ligation reaction will require the phosphate groups left on the cut plasmid, since the commercially purchasedoligonucleotides are not ordered with phosphorylation at the 5′ and 3′ ends to reduce cost.
7. Purify the BbsI-cut plasmid vector.
  a. Add DNA loading dye to the digest and separate cut vector on a 0.9% agarose gel.
  b. Gel purify the linearized vector using a gel extraction kit and elute in nuclease free water.
    **CRITICAL:** Elute the DNA in water instead of the provided elution buffer because many elution buffers in kits contain EDTA, which can interfere with ligations and downstream assays.
    ***Note:*** We use the Qiagen gel extraction kit but have achieved similar success with other commercial kits.
  c. Measure the DNA concentration of the purified cut vector using a nanodrop spectrophotometer.
  d. Dilute the cut vector to 25 ng/μL.
8. Prepare solutions for Oligonucleotide Annealing and cloning into BbsI site.
  a. Make 10× Annealing Buffer.
  b. Dilute Oligonucleotides to 100 μM in nuclease free water. Vortex to mix.
  c. Dilute the 100 μM primer stocks 1:100 in TE buffer to 1 μM final concentration (5 μL 100 μM oligonucleotide stock + 495 μL TE). Vortex to mix.
9. Prepare annealed oligonucleotides:
  a. Set up 100 μL annealing reaction in 1.5 mL Eppendorf tubes:

**Table:** 

Reagent	Amount
10× Annealing Buffer	10 μL
1 μM “top” oligonucleotide	8 μL
1 μM “bottom” oligonucleotide	8 μL
Water	74 μL
  b. Vortex, and then centrifuge briefly to bring all liquid to the bottom of the tube.
  c. Heat a beaker filled 2/3 of the way with water for 5–7 min in the microwave until boiling.
  d. While the water heats, secure the tubes containing the annealing mix into foam floats.
  e. Float the oligonucleotides in the boiling water (100°C) and place at the bench.
  f. The water will cool slowly to 25°C, annealing the oligonucleotides. Centrifuge briefly to spin down condensation and store the annealed oligonucleotides at −20°C until ready for use.
    ***Note:*** A thermocycler can be used for this step, heating at 95°C for 5 min, followed by a slow cooling ramp of 1°C/min.
10. Set up oligo ligations with 50 ng of linearized vector (not dephosphorylated).
  a. Plan for a single ligation for each annealed gRNA oligonucleotide pair, as well as a vector with no insert with and without ligase.
  b. Set up the 20 μL ligation mixes:

**Table:** 

Reagent	Amount
10× T4 Ligase Buffer	2 μL
50 ng Cut Vector (25 ng/μL)	2 μL
Annealed oligonucleotides	2 μL
Water	13 μL
T4 Ligase	1 μL
  c. Incubate ligations at 16°C for 4–5 h. Freeze ligation at −20°C or proceed directly to transformation.
    ***Note:*** Most ligases state that 15 min at 25°C is sufficient, but a longer incubation at a colder temperature yields less background. We have had success using the longer ligation described above as well as a 15 min 25°C ligation.
11. Transform ligations, including ligation negative, uncut plasmid vector positive control, and water controls, into chemically competent *E. coli* using standard protocols with 12–16h incubation.
  a. Thaw competent bacteria on ice for 10 min.
  b. Aliquot 50 μL aliquots into 1.5 mL microcentrifuge tubes.
  c. Add 5 μL of ligated DNA to each tube, and 10 ng of uncut plasmid vector positive control to the control tube. Add water to a negative control tube.
  d. Incubate on ice for 10 min.
  e. Heat shock for 45 s at 42°C.
  f. Ice shock for 2 min.
  g. Add 450 μL of sterile, antibiotic-free Luria Broth (LB) to each transformation.
  h. Incubate shaking at 37°C for 45 min.
  i. Plate using sterile technique.

***Note:*** We use NEB Stables for transformation as this *E. coli* strain has additional deleted nuclease genes that limit mutagenesis of the repetitive AAV inverted terminal repeats (ITRs).

**CRITICAL:** From the time of transformation, the protocol should be completed through Maxiprep within 10 days. Longer periods increase the chances of mutagenized ITRs, which will not allow AAV packaging, or result in death of the bacterial culture, which will prevent propagation of the plasmid.

***Note:*** NEB Stables grow more slowly than other more commonly used strains of bacteria, so they need more time to grow visible colonies, typically 20 h.

12. Select three colonies for mini prep and isolate DNA using commercial kits (e.g., QIAGEN mini prep kit).
13. Confirm successful cloning of the gRNA and intact AAV ITRs with whole plasmid sequencing.
  ***Note:*** Digests can be completed as below as an alternative to whole plasmid sequencing (Figure 2C), but may miss smaller deletions in the ITRs. Restriction digest should be used as only a rough structural check with whole plasmid sequencing being used for final verification.
  a. Digest ∼500 ng of miniprep DNA with single digests of BbsI-HF, XmaI, SnaBI-HF, and PvuII-HF in 20 μL reactions to confirm insertion of the gRNA (noncutting of BbsI) and intactness of the ITRs. Include an uncut control with no enzyme (Figure 2C).
    i. Set up the 20 μL digest mix:

**Table:** 

Reagent	Amount
Miniprep Plasmid (100 ng/μL or higher)	5 μL
10× NEB Cutsmart Buffer	2 μL
Water	Adjust total volume to 19 μL
BbsI-HF, XmaI, SnaBI-HF, or PvuII-HF	1 μL
    ii. Perform the restriction digest:

**Table:** 

Steps	Temperature	Time
Incubation	37°C	2–4 h
Heat inactivation	65°C	15 min
Hold	4°C	forever
      **CRITICAL:** Include the same miniprep control incubated with digest buffer but no enzyme. Because DNA migrates higher in the gel when it is cut by restriction enzyme, this control confirms the BbsI sites can no longer be cut.
      ***Note:*** In rare cases, some gRNAs will retain the BbsI cut site. This can be identified when building the plasmid files in Step 4.
      **CRITICAL:** The XmaI, SnaBI, and PvuII digests cut within the ITR region that would be packaged in the AAV. If the restriction digest does not look like the predicted digest from SnapGene or Benchling, that plasmid is unlikely to be correctly packaged into AAV as the ITRs are mutated. That culture should be discarded and a different culture should be selected for downstream analysis. An example of a digest following ITR mutation is shown in Figure 2.
14. From the digest or sequence-verified culture, grow a 250 mL maxiprep culture in LB with 100 μg/mL Ampicillin for at least 20 h.
  a. Purify with a maxiprep purification kit.
  b. Record the concentration of the final AAV-CRISPR plasmid and store at −20°C until AAV transfection.
15. Sequence to confirm gRNA is present in the final plasmid using either whole plasmid sequencing (no primer required) or targeted sequencing using this primer:

**Figure 1:**
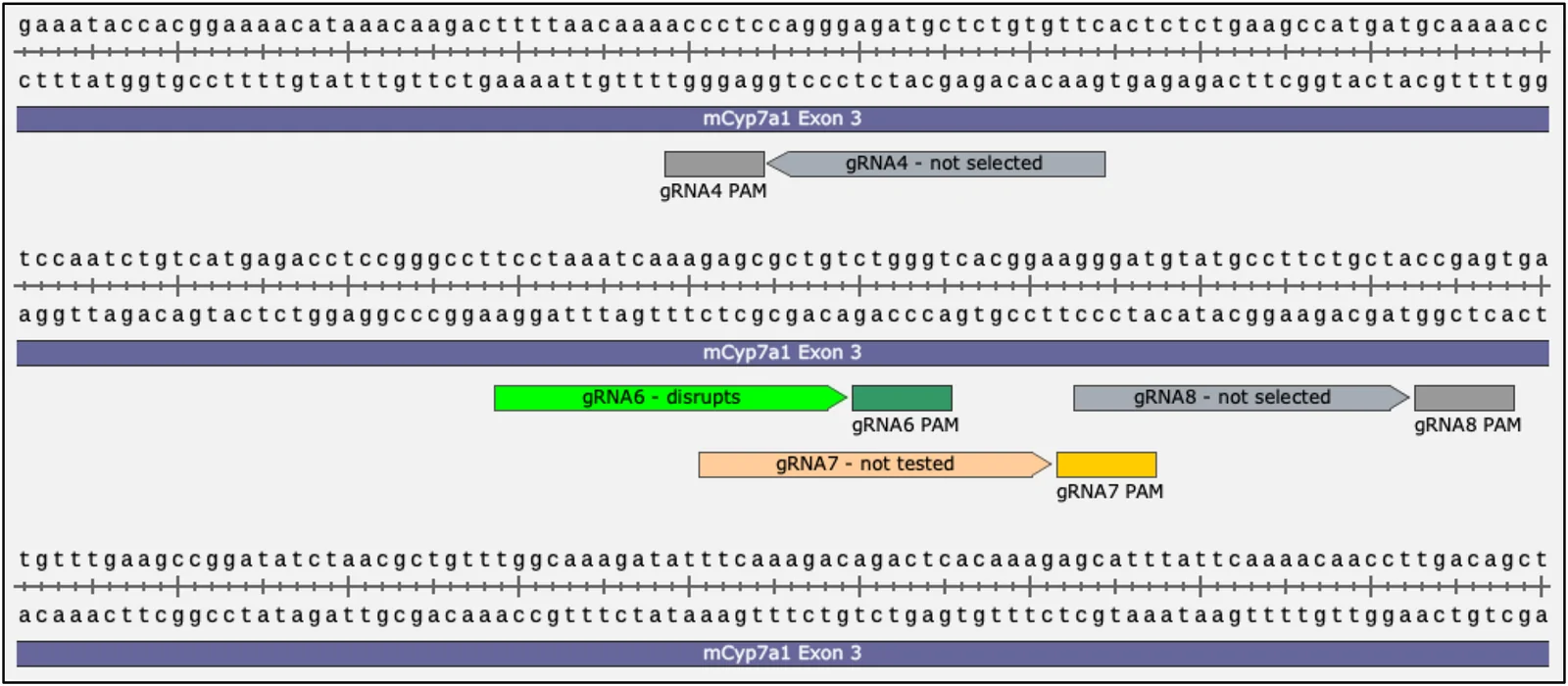
Annotation of DNA for gRNA design.

**Figure 2:**
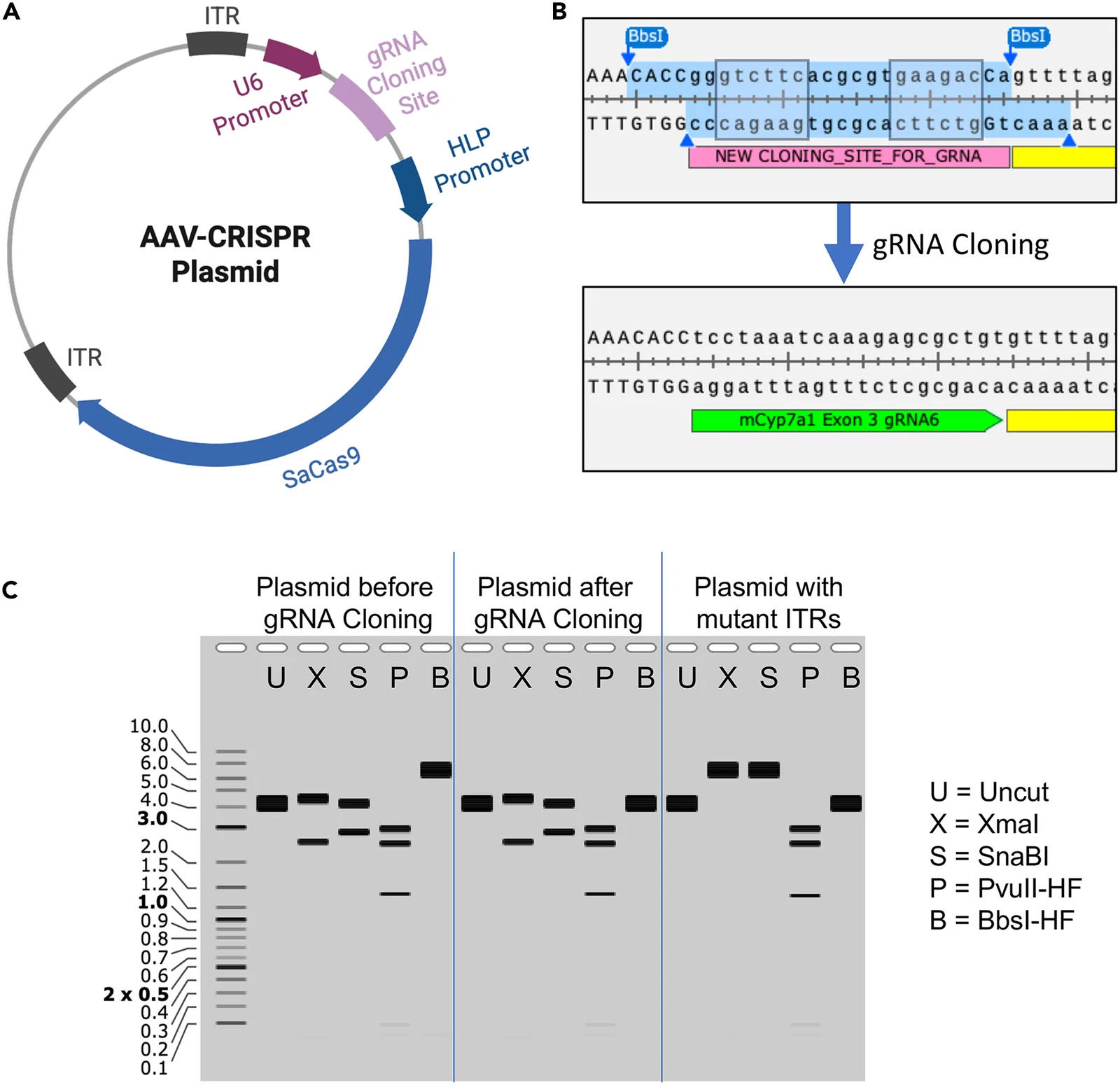
gRNA cloning and plasmid confirmation (A) Plasmid diagram based on Addgene #109314.^1^^,^^8^ (B) gRNA cloning with the BbsI restriction enzyme. The BbsI binding sites are highlighted inside of the boxes. (C) Example digests with Uncut (U), XmaI (X), SnaBI (S), PvuII (P), and BbsI (B). Following gRNA cloning, BbsI should no longer cut in most cases. The X, S, and P digests are used to confirm presence of the AAV inverted terminal repeats (ITRs). If the ITRs are damaged, the CRISPR system cannot be packaged into AAV. Whole plasmid sequencing is recommended for final vector gRNA insert and ITR confirmation.

U6forseq: CCTTCATATTTGCATATACGATACAAGGCTGTTAG.

**Pause point:** The protocol can be paused at this point indefinitely.

### Production of adeno associated virus

**Timing: 3–4 weeks depending on preparation method**

Preparation of Adeno Associated Virus (AAV) is time consuming and involves multiple complex steps including a triple transfection method, cesium chloride gradient ultracentrifugation, and viral titer calculations.^19^ We recommend having the AAVs produced commercially using either academic core facilities or commercial sources.

16. Select at least two of the AAV-CRISPR plasmids for production of Adeno Associated Virus (AAV) serotype 2/8.
17. Request production from a core company, and obtain the AAV.

***Note:*** Typically, 100–200 μg of the AAV-CRISPR plasmid is necessary for production. The goal is to obtain at least 1×10^13^ genome copies of virus, which is enough for 20 mice.

***Note:*** AAVs are made in adherent HEK293T cells in 15-cm plates transfected with packaging plasmids pAdDeltaF6 (Addgene #112867) and pAAV2/8 (Addgene #112864) and transfer plasmids as generated in Steps 6–15. We pool 20–40 plates per preparation and perform a single cesium chloride gradient spin followed by dialysis and qPCR titer.^19^

**Pause point:** The protocol can be paused at this point until the *in vivo* testing can be performed.

### Primer design for quantification of DNA editing

**Timing: 2 h**

The goal of this section is to design primers to amplify a ∼400 bp region surrounding the gRNA cut site. These primers are used for both DNA-SV qPCR and Sanger sequencing analysis for INDEL analysis and can be annotated on the gRNA design files generated in Steps 1–4.

18. Open the genomic DNA file with annotations for the gRNAs that were designed in Steps 1–4.
19. Highlight the area 300 bp upstream and 300 bp downstream of the gRNA site.
20. Input this 622 bp sequence into NCBI Primer Blast and select the following parameters:
  a. Select minimum 375 and maximum 450 bp for PCR Product Size.
  b. Set database to, “Genomes for selected eukaryotic organisms,” and input “mus musculus.”
  c. Select “Show results in a new window,” and initiate via “Get Primers.”
21. Select primer pairs that are at least 125 bp away from the gRNA site on each side which have high specificity.

***Note:*** Consider the predicted size of each off-target amplicon – a 3 kb will not easily amplify under fast qPCR conditions, but 300 bp off targets more likely will amplify if the primer matches well.

22. Choose two different specific primer sets that do not overlap in binding site to maximize amplification of the target region. Use the following selection criteria:
  a. Avoid repetitive regions, especially 4 or more of same base.
  b. No more than 3 out of the last five 3′ bases should be C or G.
  c. Target a T_m_ 57°C–62°C with GC content about 50%.

***Note:*** If no suitable primers return, increase the window for DNA bases selected from the genomic DNA sequence. Alternatively, primers can be designed by hand but should be verified for specificity using Primer BLAST.

23. Test the primers for efficiency using the standards and setup for the qPCR protocol below (Steps 75–76).
24. Order oligonucleotides for testing the gRNAs which will be tested via AAV.

***Note:*** Oligonucleotides can be ordered from any commercial company, such as Integrated DNA Technologies or Sigma Aldrich.

**Pause point:** The protocol can be paused at this point indefinitely.

### *In vivo* pilot experiment to test AAV-CRISPR

**Timing: 10–14 days**

This section describes the design and execution of the *in vivo* pilot experiment to test the effectiveness of the newly prepared AAV-CRISPR to disrupt the gene of interest. It includes considerations for experimental design, as well as notes on mouse strain, sex, and route of AAV administration.

25. Design the CRISPR tester experiment with at least n=3 mice/group, including a control group (Figure 3A).

***Note:*** It is helpful to have more control mice than experimental mice as the increased number of control animals allows for tighter gene editing analysis. We often use n=5–6 mice for the control group due to the need for efficient control amplification for later DNA qPCR analysis.

***Note:*** This protocol has been optimized for C57BL/6J mice. It may be applicable to other mouse strains but testing AAV transduction using a fluorescent reporter such as GFP should be used to determine dosing required for efficient liver targeting.

**CRITICAL:** Mice should be at least 8 weeks old at time of AAV-CRISPR testing. Gene editing with AAV-CRISPR occurs over a period of days to weeks. In younger animals, the liver has a larger proportion of dividing hepatocytes. Since episomal AAV genomes are lost or diluted with cell division, this can lead to lower editing rates in younger animals.

26. Prepare AAV-CRISPR and PBS control doses in a biosafety cabinet.
  a. Dilute AAV to 5×10^11^ genome copies per 100 μL of sterile PBS in a biosafety cabinet.
  b. Prepare 10% excess to account for the void volume in syringes.
  c. Prepare an adequate amount of PBS with no virus for the control injections.

***Note:*** Adeno associated virus is typically classed at biosafety level 1. Wipe surfaces with 10% bleach solution followed by 70% ethanol. Dispose of all materials that have contacted virus in biohazard waste.

27. In the mouse facility, prepare a syringe with 100 μL of the virus or PBS to be injected.
28. Identify the mouse and administer the prepared virus via intraperitoneal injection (Figure 3B).
  a. Use a scruff restraint, ensuring the mouse is held securely and safely.
  b. Deliver the virus in the lower left quadrant, ensuring that both the skin and abdominal wall have been punctured with the needle.
  c. Pull back on the syringe to ensure no blood vessels have been punctured.
  d. Remove the syringe and place in a biohazard sharps container.
    ***Note:*** With the mouse still restrained, inspect the abdomen to inspect the injection site for subcutaneous swelling. If swelling is observed, this suggests that the AAV was delivered between the abdominal wall and skin and the liver will not be transduced with AAV, constituting a failed injection.
    ***Note:*** Intraperitoneal injections are very efficient for AAV2/8 delivery to the liver.^20^ Intravenous injections via the tail vein or retroorbital plexus, which are more difficult to perform can also be used, but are not necessary for this AAV serotype. Dosages for other injection routes and serotypes may need to be optimized.
    ***Note:*** It has been previously reported that AAV delivery is less efficient in female mice,^21^^,^^22^ but this is contrary to our experience where we still achieve efficient AAV delivery to the liver. For 8-week old female mice that have not been exposed to males, most are not yet in estrous cycling and can be injected by intraperitoneal injection like males of the same age. For older females, or females which have been confirmed to be cycling, we inject the day after estrous and observe efficient AAV delivery.
  e. Return the mouse to its cage and complete observations at 24, 48, and 72 h, including a cage change at 72 h post-injection.
    ***Note:*** We change cages 72 h after AAV injection as most virus is cleared from the body by that time. This allows disposal of any potentially infective virus that may have been shed.
29. Ten to fourteen days after AAV-CRISPR injection, harvest the liver for editing analysis (Figure 3C).
  a. Anesthetize and euthanize mice following approved methods of euthanasia.
    ***Optional:*** Bleed mice to clear the liver of blood, then proceed to euthanasia by cervical dislocation and thoracotomy.
  b. Dissect open the abdominal cavity and remove the whole liver by grasping the connective tissue that joins all the lobes and excising surrounding tissue away.
  c. Excise the largest lobe into several small pieces, estimating the size between 20–50 mg. Place the pieces around the inside of a microcentrifuge tube so that they are not touching and snap freeze in liquid nitrogen (Figure 3C).
    ***Note:*** Placing the tissue pieces into the tube so it is not touching allows for selection of pieces for DNA, RNA, and possibly protein analysis without re-cutting frozen tissues.
  d. Snap freeze remaining liver tissue in a separate tube.
  e. Store the collected liver tissue at −80°C until analysis.
    **Pause point:** The protocol can be paused at this point until DNA analysis is performed.

**Figure 3:**
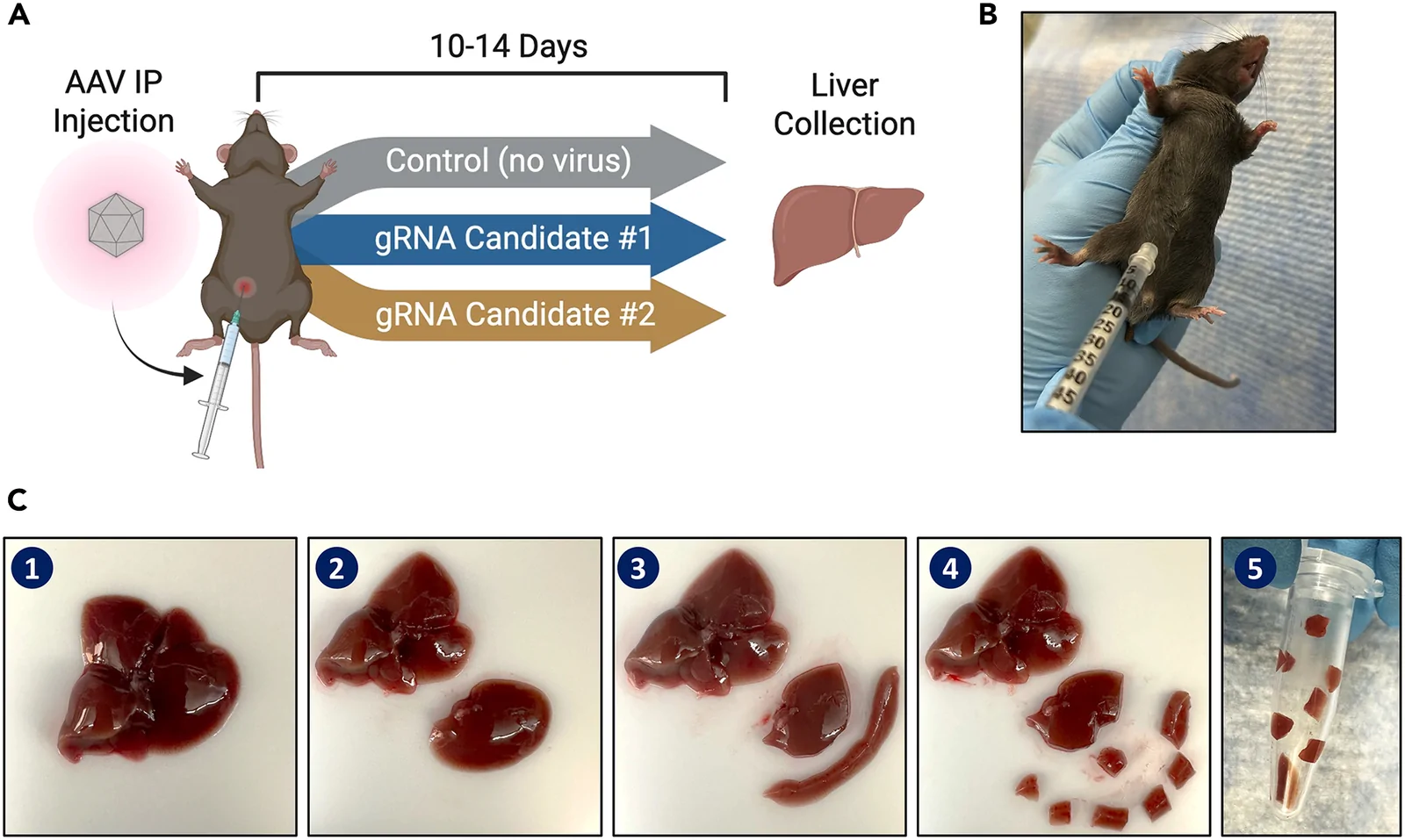
*In vivo* testing of AAV-CRISPR (A) Experimental design of a pilot AAV-CRISPR experiment. At least two gRNAs are recommended for testing of each new target. (B) Intraperitoneal injection. Mice are restrained by scruffing and then 100 μL of sterile phosphate buffered saline (PBS) containing 5×10^11^ genome copies of AAV are injected intraperitoneally in the lower left quadrant of the mouse’s abdomen. (C) Liver collection. The largest lobe is excised from the liver and the outer portion of the lobe is cut into pieces. The liver pieces are placed on the side of a microcentrifuge tube without touching and then are snap frozen. The pieces can be retrieved later without the need to cut new liver piece.

### DNA extraction for assessment of *in vivo* gene editing events

**Timing: 6 h**

The goal of this section is to extract genomic DNA from the livers of mice treated with AAV-CRISPR for downstream analysis of editing events. We use the Qiagen DNeasy kit for liver DNA extraction but have modified the protocol to increase volumes in the initial steps. Similar modifications may work for other commercial kits but should be optimized for use in each case. All steps shown here were optimized for the Qiagen DNeasy kit.

30. Homogenize a 20–30 mg piece of liver in 360 μL ATL buffer using 4 ceramic beads in a beadruptor on the quick cycle for 15 s at 4 m/s. Briefly centrifuge in a benchtop centrifuge at 9500 × *g* to bring the liquid back to the bottom of the tube.

***Note:*** If a beadruptor is not available, pulverize the liver sample with a dounce homogenizer or finely mince with scissors before proceeding. Other methods will require increased time in Step 32.

***Note:*** Additional ATL, AL, and proteinase K buffer can be purchased separately from Qiagen.

31. Add 40 μL of the Proteinase K in the kit and vortex to mix.

***Note:*** If Proteinase K from the kit is not available, use an equal volume of a 10 mg/mL Proteinase K solution.

32. Incubate samples 20–30 min at 50°C following homogenization with occasional vortexing.
33. Add 400 μL Buffer AL and vortex to mix.
34. Add 400 μL 100% ethanol and vortex to mix.
35. Pipette 700 μL of the sample mixture into a spin column with collection tube. Centrifuge at 9500 × *g* for 1 min. Discard flow through.
36. Apply the remaining samples from Steps 30–34 to the spin column. Centrifuge at 9500 × *g* for 1 min. Discard flow through and collection tube.
37. Place the spin column in a new collection tube. Add 500 μL of Buffer AW1. Centrifuge at 9500 × *g* for 1 min. Discard flow through and collection tube.
38. Place the spin column in a new collection tube. Add 500 μL of Buffer AW2. Centrifuge at 9500 × *g* for 3 min. Dump flow through.
39. Centrifuge again at 9500 × *g* for 3 min to dry the membrane.
40. Transfer the spin column to a clean 1.5 mL Eppendorf tube. Add 250 μL nuclease free water. Let column stand for 5 min.

**CRITICAL:** Elute in water only. Do not use the elution buffer in the kit as it can interfere with future qPCR applications.

41. Centrifuge again at 9500 × *g* for 2 min to elute DNA.

**CRITICAL:** From this point on, work with the DNA on ice.

42. Measure the DNA concentration using a nanodrop spectrophotometer.

***Note:*** Ideally most samples will be over 300 ng/μL with an A260/A280 ratio close to 2.

**CRITICAL:** The average of the control samples must be over 200 ng/μL for generation of a dilution series to make a standard curve below.

43. Separate DNA for the 1:3 dilution DNA standard curve. This standard curve will be used in later steps 72–75.
  a. Dilute DNA from individual Control livers to 200 ng/μL to a final volume of 100 μL and measure the concentration to confirm it is between 195 and 205 ng/μL.
  b. Label 1 through 8 on the lids of an 8-well PCR strip tube.
  c. Mix equal volumes of the 200 ng/μL control DNAs to generate 300 μL of Pooled Control DNA at 200 ng/μL in the first well of a PCR strip tube.
  d. Add 200 μL of nuclease free water to the remaining 7 tubes in the strip.
  e. Seal Tube 8, which will be used as a water negative control.
  f. Use 100 μL of the pooled DNA sample in the first tube to do a serial 1:3 dilution in tubes 2–7.
44. Prepare hepatic DNA for downstream analysis.
  a. Dilute DNA from all mice in the experiment to 25 ng/μL in at least 250 μL aliquots.
  b. Store remaining undiluted DNA at −20°C in case additional future dilutions are needed.
45. Store DNA samples at −20°C until analysis.

**Pause point:** The protocol can be paused at this point indefinitely.

### Analysis of AAV genomes in murine liver

**Timing: 3 h**

This section describes qPCR-based quantification of AAV genome copies in the liver. Using genomic DNA purified from mouse liver, we determine the number of genome copies per microgram of DNA is determined by qPCR to confirm efficient delivery of AAV to the livers of mice treated with the AAV-CRISPR.

46. Obtain primers specific to sequences present on the AAV delivered to the liver.

***Note:*** Primers should be selected for specificity to a common region across similar plasmids, such as a promoter region or product that is present on all plasmids used. The primers used in this case are specific to SaCas9 (Figure 4A):

**Figure 4:**
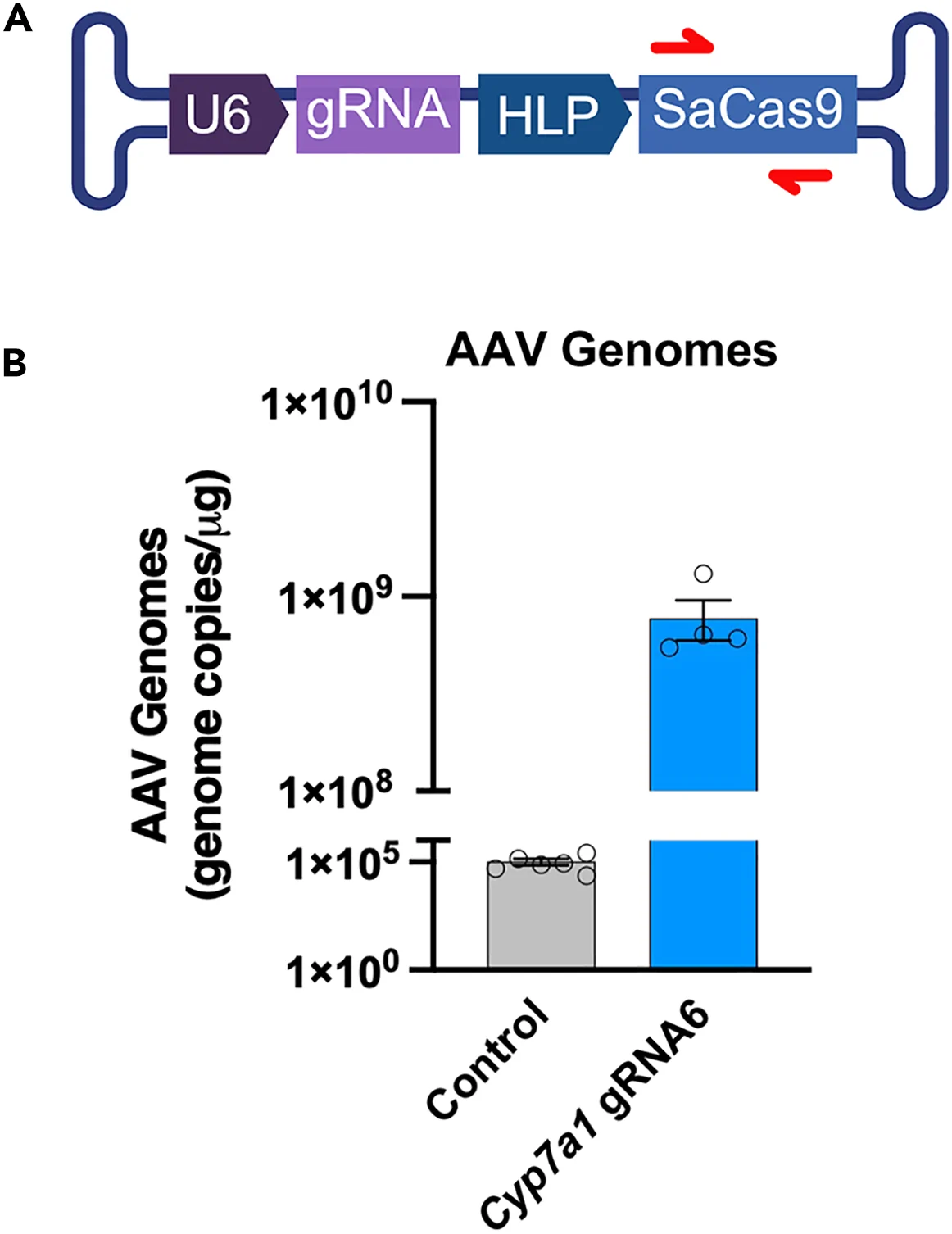
Assessment of hepatic AAV genomes (A) AAV-CRISPR diagram showing the location of the SaCas9 titering primers. (B) Hepatic AAV genomes in Control mice treated with PBS alone compared to mice injected with 5×10^11^ genome copies of an AAV-CRISPR expressing *Cyp7a1* gRNA6. Error bars are standard error of the mean (SEM). n = 4-6 mice/treatment.

SaCas9 Forward: CCGTCGTGAAGAGAAGCTTCATC.

SaCas9 Reverse: CCACCTCATAGTTGAAGGGGTTG.

47. Prepare plasmid standards from the plasmid used to make AAV (e.g., Addgene #109314).^8^
  a. Label 1 through 8 on the lids of an 8-well PCR strip tube. Add 180 μL of water to tubes 2–8 of a PCR strip tube and seal tube 8 to be used as a water control.
  b. Dilute 250 μL of plasmid DNA to 1 ng/μL into the first well of the PCR strip tube.
  c. Do a serial dilution (1:10) using 20 μL of the plasmid DNA in tube 1, leaving tube 8 as a water-only control. The first dilution is 0.1 ng/μL.
48. Prepare enough qPCR mix for 10 μL reactions for all samples and standards in triplicate with 20% extra to account for pipetting error. Make separate qPCR mix for each primer set.

**Table:** 

Reagent	Amount (1×)
2× SYBR Mix	5 μL
Primer 1 (100 μM)	0.0375 μL
Primer 2 (100 μM)	0.0375 μL
Water	0.925 μL
Diluted DNA Template (25 ng/μL)	4 μL

***Note:*** These steps have been tested using Roche Lightcycler 480 2× PCR mix and Azureblue 2× PCR mix on a Roche Lightcycler 480 qPCR machine. Reaction conditions should be optimized for other reagents and instruments.

49. Prepare the qPCR plate.
  a. Pipet 4 μl samples, standards, blanks into a 384-well qPCR plate in triplicate.
  b. Apply 6 μl of qPCR mix to the appropriate rows on the plate.
  c. Seal with optically clear tape.
  d. Centrifuge (1000 × *g* for 2 min at 25°C) to remove bubbles.
50. Set up the qPCR instrument (such as Roche Lightcycler 480 used here).
  a. Define sample, blank, and standard locations on the plate as well as the zones each primer set has been used.
  b. Input the concentration of standards from the 1:10 dilution.
  c. Run using a standard qPCR program with a melt curve, such as:

**Table:** 

Steps	Temperature	Time	Cycles
Initial Denaturation	95°C	5 min	1
Denaturation	95°C	15 sec	40 cycles
Annealing	60°C	10 sec	
Extension	72°C	10 min	
Pre-melt Denaturation	95°C	10 sec	1
Melt Curve	60°C–97°C	2.5 °C/sec	1
Hold	10°C	hold

51. Confirm the standard curve looks appropriate.
  a. Efficiency should be close to 2, which is equivalent to 100%. This is a slope of −3.33.
  b. The melt curve should show a single product.
52. Calculate the vector genomes per microliter equivalent in the plasmid standard curve. See, “Table S1 AAV Genomes Template.xlsx.”
  a. Adjust plasmid size to estimated formula weight by taking the bp size of the plasmid and multiplying by 662. For Addgene #109314,^8^ this is 6967 bp × 662 = 4,612,154 g.
  b. Molarity = [Starting Standard Concentration in ng/μL] × 1×10^6^ (converting grams to nanograms) × (1/FW).
  c. Convert to genome copies: Molarity × 6.022 × 10^23^ particles/mole × 1 × 10^3^ (converting Liters to mL) = genome copies per mL (GC/mL).
53. Use the GC/mL for your starting standards dilution (0.1 ng/μL) and calculate the GC/mL for each dilution. Convert this to Log10.
54. Graph the crossing point values from the qPCR (or cycle threshold depending on the system) versus the Log10 Quantity and do linear regression to generate the equation of the line, y=mx+b.
  a. The slope should be near −3.33.
  b. The y intercept should be around 42–45.
  c. Aim for R^2^ of 0.97 or better.
55. Using the equation of the line, calculate the genome copies per sample, and then adjust to GC/μg of DNA (Figure 5B).
  a. Because 100 ng of DNA were loaded at the qPCR step, divide by 100 to convert to GC/ng of liver DNA.
  b. Multiply by 1000 to convert to GC/μg of liver DNA.

**Figure 5:**
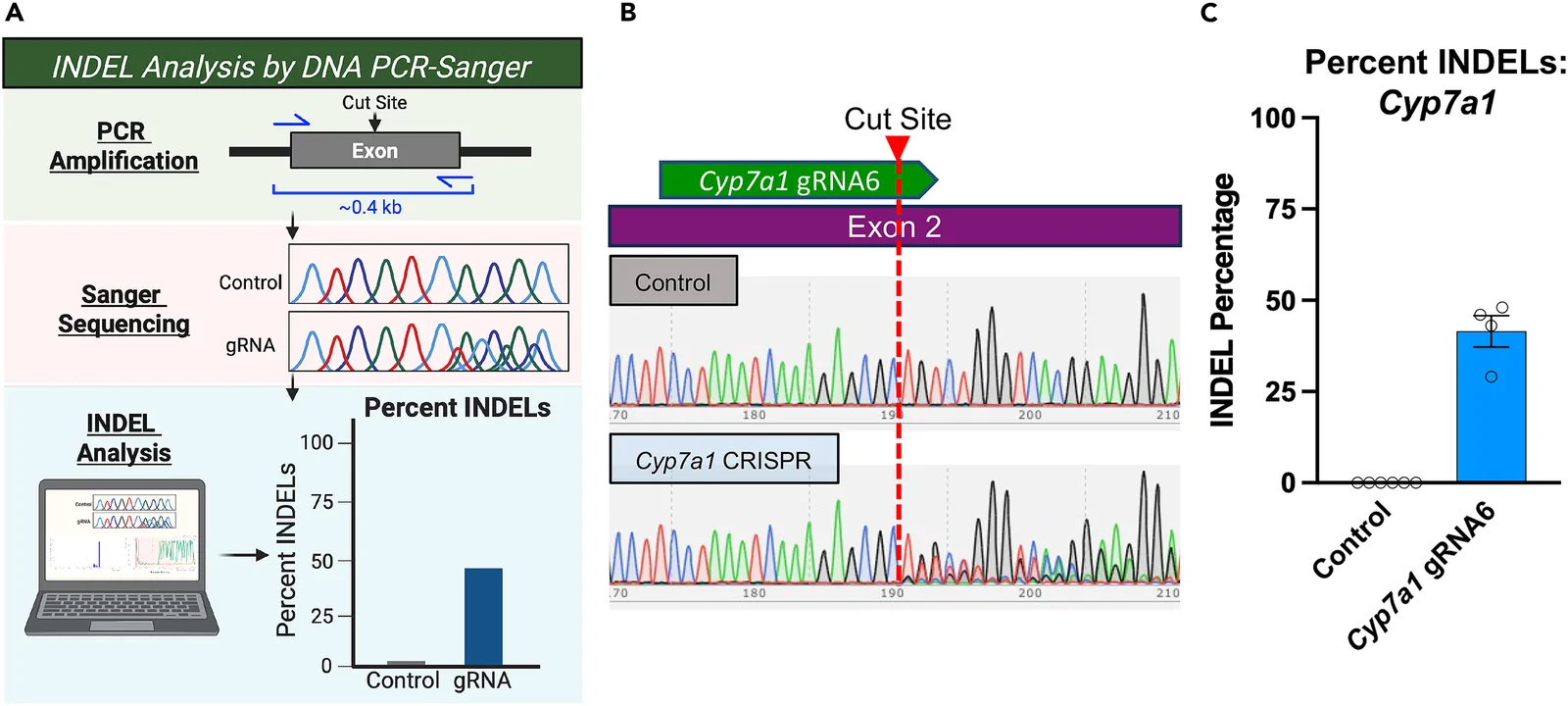
Hepatic AAV-CRISPR INDEL analysis (A) Overview of AAV INDEL analysis. The target region for CRISPR editing is amplified by traditional PCR with a high-fidelity polymerase and gel purified. Sanger sequencing is completed and total INDELs are assessed using online computational tools. (B) Liver DNA sequence traces from Control mice treated with PBS alone compared to mice injected with 5×10^11^ genome copies of an AAV-CRISPR expressing *Cyp7a1* gRNA6. The primer directionality for sequencing is from left to right, and after the cut site, the *Cyp7a1* CRISPR sample shows disorganized traces indicative of INDELs. (C) Liver INDELs in Control mice treated with PBS alone compared to mice injected with an AAV-CRISPR expressing *Cyp7a1* gRNA6. Error bars are standard error of the mean (SEM). n = 4-6 mice/treatment.

***Note:*** Mice that received no virus injection should have less than 10^6^ GC/μg while mice receiving CRISPR should have greater than 1×10^8^ genome copies per μg for a successful CRISPR delivery. Mice with lower vector genomes likely had inefficient or failed AAV delivery should be excluded from later analysis.

***Note:*** Mice with long-term delivery of AAV-CRISPR (20 weeks or more) will have lower AAV genomes due to slow turnover of hepatocytes and loss of AAV episomes. We recommend comparing to saline/ no AAV treated animals to determine the expected AAV levels.

***Note:*** Low detection of AAV genomes can be a result of failed injections, when AAV is accidentally delivered between the abdominal wall and skin. Therefore, n=3 or more mice are used for test experiments with the goal of getting at least 2–3 successful AAV deliveries per gRNA for analysis.

### Assessment of insertions and deletions in the liver

**Timing: 6 h**

This section describes the steps required to assess small DNA insertions and deletions (INDELs) in the genomic DNA of mice treated with AAV-CRISPR (from steps 30–45). The analysis involves PCR, followed by gel extraction and Sanger sequencing. Gene disruption by AAV-CRISPR leads to discordant sequence traces compared to controls, which can be quantified using online tools such as EditCo Inference of CRISPR Edits (ICE) or Tracking of INDELs by Decomposition (TIDE).^2^^,^^3^

**CRITICAL:** Before doing DNA analysis for insertions and deletions (INDELs), validate the primers designed in steps 18–24 using a standard curve to ensure they have good amplification efficiency and amplify a single product. See Step 75 below.

56. Pipette 2 μL of the 25 ng/uL genomic DNA into strip tubes.

**CRITICAL:** It is important to pipette the DNA first, as high fidelity polymerases will degrade the primer DNA if left for too long. Complete DNA addition before preparing the master mix.

57. Set up PCR Master Mix on ice, adjusting if more than 8 samples are to be run:

**Table:** 

Reagent	Amount (8 reactions)
Water	180 μL
5× Reaction Buffer (Kapa HiFi Kit)	45 μL
10 mM DNTPs	6.75 μL
Primer 1 (100 μM)	1 μL
Primer 2 (100 μM)	1 μL
DMSO	6.75 μL
Polymerase (Kapa HiFi)	1 μL
DNA (25 ng/μL, in tube in above step)	2 μL

58. Vortex briefly and pipette 25 μL of the master mix into each well of the strip for a total volume of 27 μL. Briefly centrifuge to remove bubbles and ensure all solutions are at the bottom of the tube.
59. Run the PCR protocol in the thermocycler:

**Table:** 

Steps	Temperature	Time	Cycles
Initial Denaturation	95°C	2 min	1
Denaturation	95°C	30 sec	30–35 cycles
Annealing	60°C	30 sec	
Extension	72°C	1 min	
Final extension	72°C	3 min	1
Hold	10°C	hold

60. Following PCR, cast a 0.8% agarose gel with ethidium bromide. Plan to load 35 μL of sample and leaving an empty well between adjacent samples.
61. While the gel sets, clean the gel chambers and prepare fresh 1× TAE running buffer to fill the chamber.
62. Add 6× loading dye to the DNA at a final concentration of 1× and mix well before loading.
63. Load the samples, leaving an empty well between every sample and between the ladder and samples.

**CRITICAL:** Skipping a lane helps avoid contamination between samples, which minimizes contamination of controls with edited DNA and is very important for downstream analysis.

64. After the gel has run, use a clean scalpel or gel tweezers and an ultraviolet lamp or gel box to excise the ∼400 bp products, decontaminating the blade between samples and limiting the exposure of the sample to ultraviolet light.
  a. Cut bands with little margin to minimize extra agarose.
  b. Transfer each gel slice to a clean 2 mL microcentrifuge tube.
65. Use a gel extraction kit to purify the DNA from the gel pieces.

***Note:*** Most commercially available kits, including the Qiagen Gel Extraction Kit, require weighing of the agarose piece and using 3 gel volumes of a buffer to dissolve the agarose. In our experience, it is not necessary to use varying amounts of reagent for each sample so long as the gel pieces are within 100 mg of each other. Standardize weight of the heaviest piece and set 3× that volume for all samples.

66. Elute the DNA using 50 μL of nuclease free water.

***Note:*** Do not use buffered solutions or the kit supplied elution buffer as it can interfere with some enzyme reactions.

***Note:*** Less elution buffer can be used if there is concern of low DNA yield.

67. Measure A260/A280. Samples should have concentrations close to 10 ng/μL or higher and have A260/280 ratio close to 2.
68. Select one of the two primers used for DNA-SV qPCR above for sequencing.
  a. Check upstream of the gRNA to make sure there are not low-complexity sequences, including homopolymer runs or tandem repeats.
  b. Ensure the primer selected is more than 125 bp from the cut site.
69. Prepare samples for sequencing based on the instructions from the sequencing company and send for analysis.

#### INDEL analysis

The analysis performed here uses EditCo Inference of CRISPR Edits (ICE) Analysis (Figure 5A). This analysis is formerly from Synthego^3^ and now hosted at EditCo, and was adapted from the original TIDE^2^ analysis. Either tool can be used for this analysis, which uses the discordance between the control and edited DNA sequences from the liver DNA amplicons to calculate editing efficiency. The protocol here utilizes EditCo ICE, which allows for batch upload.

70. Download your sequencing data as ABI files and first align them to the genome to see visually if editing occurred (Figure 5B).
  a. In SnapGene, open the genomic DNA file for the target gene from steps 1–4. Save a new copy into the experimental folder you are working in.
  b. Navigate to Tools → Align to Reference DNA Sequences → Align Imported Sequences.
  c. Select your sequencing files and click open.

***Note:*** Control samples should have perfect alignment with clean peaks.

***Note:*** Experimental samples with successful gRNAs should be clean upstream of the cut site and show overlapping peaks with more difficulty calling bases following the cut site (Figure 5B).

***Note:*** The directionality of the primer used for sequencing is sometimes the reverse primer, so sequencing traces may run from right to left.

71. Set up EditCo ICE^3^ analysis: https://www.editco.bio/crispr-analysis.
  a. Name the sample based on the sample ID being assessed, for example, Experiment1_Cyp7a1_gRNA2_05.
  b. For nuclease, select SpCas9 even if SaCas9 is being used.
    ***Note:*** EditCo ICE does not currently offer functionality specific to SaCas9, but selecting SpCas9 will still yield the editing results. The cut site will still be captured, and editing analysis will still be completed.
  c. Paste the gRNA in 5′ to 3′, and do not include the PAM motif. Remember to paste the reverse complement if the gRNA is on the bottom strand of the DNA.
  d. Drop in the control.ab1 file and the experimental.ab1 file to be analyzed and add the samples to the analysis.
  e. Once all samples are added, click Analyze.
    ***Note:*** If several samples are used, there is a batch upload option.
72. Look at the editing data visualization to assess whether on-target editing occurred. Editing efficiencies of greater than 30% via ICE analysis are indicative of a functional gRNA.
73. Export the data. If using EditCo ICE, an email address will need to be provided. Add these data to an editing analysis summary Excel sheet.
74. Graph the percent INDEL data (Figure 5C).

### Assessment of hepatic DNA-level structural variants

**Timing: 3 h**

This section describes a qPCR-based method that captures DNA structural variants (SVs). Measuring DNA-SVs on liver DNA using qPCR takes advantage of the inability of most polymerases to amplify large, multiple kilobase DNA inserts in a short period of time. Thus, there is a reduction in the amplification of the DNA SV region when compared to non-edited amplification of the same region, which can then be used to quantify a percentage of DNA structural variant events, including AAV integrations (Figure 6A).

75. Plan the qPCR plate layout for samples to be tested including sections for new primers. To measure DNA-SVs, a primer set that amplifies in a non-edited region also should be included.

**CRITICAL:** Test primer sets using the 1:3 standard curve DNA prior to running samples to ensure primers are selected which are specific and have appropriate amplification specificity.

***Note:*** We use Internal Positive Control standard primers from Jackson Laboratories genotyping protocols as the “housekeeping” or non-edited control region. The amplification from these primers serves as the housekeeper for total amount of DNA in the same way that housekeeping genes do for qPCR from cDNA. We use the following primer sets:

**Figure 6:**
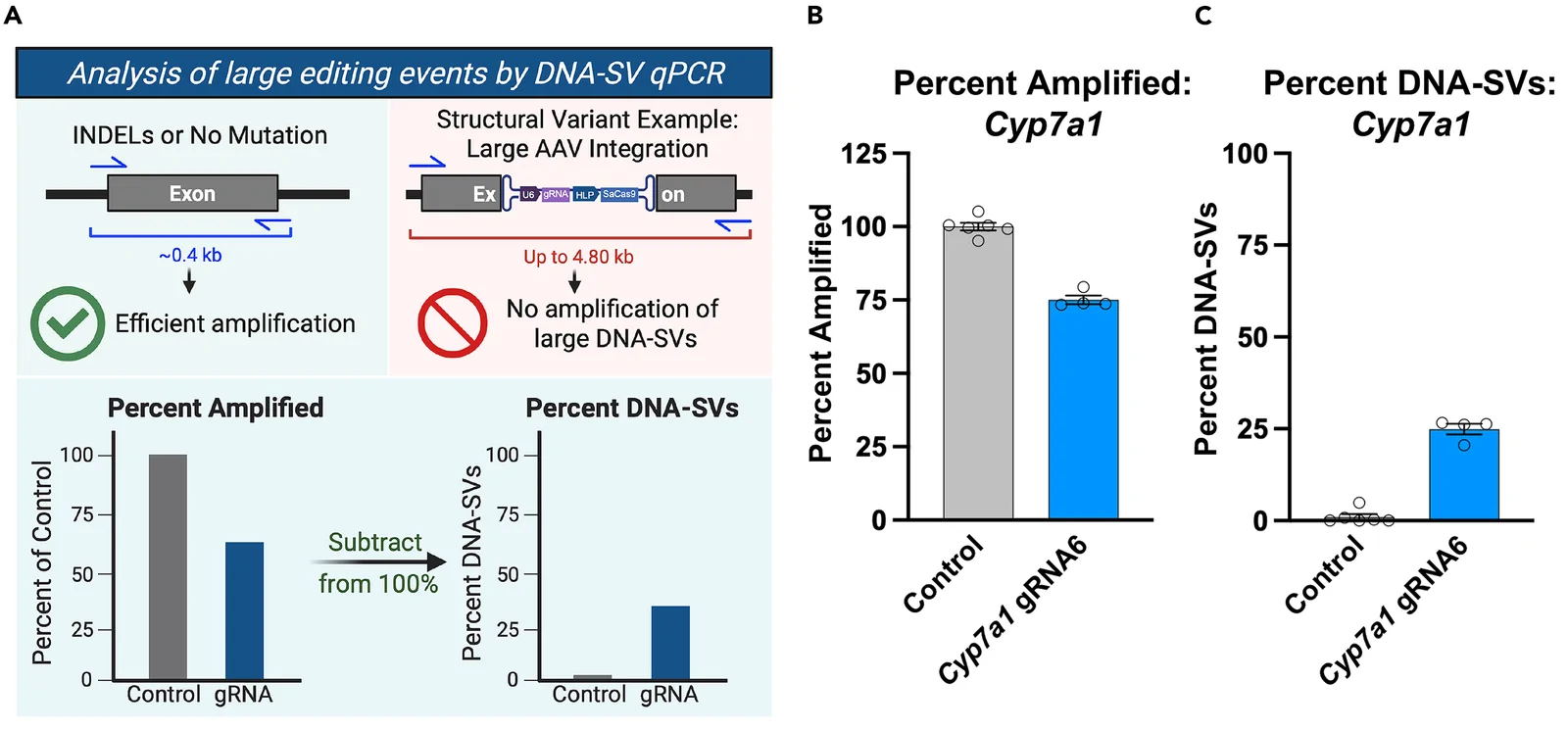
Hepatic AAV-CRISPR DNA-SV analysis (A) Overview of DNA-SV analysis, which includes the frequent AAV integrations associated with AAV-CRISPR. The target region for CRISPR editing is amplified by DNA qPCR. While the 400 bp target region is easily amplified, large DNA-SVs such as AAV genome integrations cannot be amplified. This reduction in amplification can be converted to percent integration for an indirect report of DNA-SV events. (B) CRISPR target region amplification in Control mice treated with PBS alone compared to mice injected with an AAV-CRISPR expressing *Cyp7a1* gRNA6. The *Cyp7a1* CRISPR samples have decreased amplification due to DNA-SV events. (C) Percent AAV integration in Control mice treated with PBS alone compared to mice injected with an AAV-CRISPR expressing *Cyp7a1* gRNA6. The *Cyp7a1* CRISPR samples have approximately 25% DNA-SVs. Error bars are standard error of the mean (SEM). n = 4-6 mice/treatment.

Control Forward: AGT GGC CTC TTC CAG AAA TG.

Control Reverse: TGC GAC TGT GTC TGA TTT CC.

76. Prepare the qPCR reactions as in Steps 46–51 with the following changes:
  a. Use the 1:3 dilution Control Liver DNA Standard Curve made in Step 43.
  b. Each sample will need to be assessed using the Internal Positive Control housekeeping primers (Step 75) as well as primers specific to the CRISPR editing site (designed in Steps 18–24, tested in Step 75.
  c. The Standard Curve should be run for each primer set, which should have a slope close to −3.3 and an efficiency close to 2.
  d. Export the data as concentration based on the standard curve.
77. Analyze the qPCR data using standard qPCR analysis that would be used for gene expression, normalizing to the Internal Positive Control housekeeping primers and setting the Control liver DNA samples to 100% (Figure 6B, see also “Table S2 DNASV Calculation Example.xlsx”).
78. Convert to percent of control DNA and then subtract from 100. This is the DNA-SV Percentage (Figure 6C).
79. Add these percentages to a summary sheet with the INDEL data and graph the DNA-SV percentages (Figure 6C).

***Note:*** The DNA-SV Percentage should be greater than 25% for efficient gRNAs.

***Note:*** For examples of DNA-SVs, AAV integrations, which can be over 4 kb, are so large that the polymerase cannot amplify across the CRISPR cut site. In addition, very large deletions or translocations at the target locus would also fail to amplify. This loss of amplification appears as a “decrease” in DNA, which is reflective of the loss of amplification from large AAV integrations, deletions, and translocations. This percentage, when subtracted from 100, is termed percent DNA-SVs, incorporating these editing events.

### Quantification of measurable editing events

**Timing: 15 min**

This section combines the INDEL analyses with the DNA-SV measurements to calculate total editing events. When testing multiple gRNAs, this analysis allows for direct comparison of the efficiencies of different gRNAs.

80. Generate another graph with INDEL and DNA-SVs stacked on top of each other (Figure 7B). These summed edits can be used to determine the most efficiently disruptive gRNA tested (Figure 7C).

**Figure 7:**
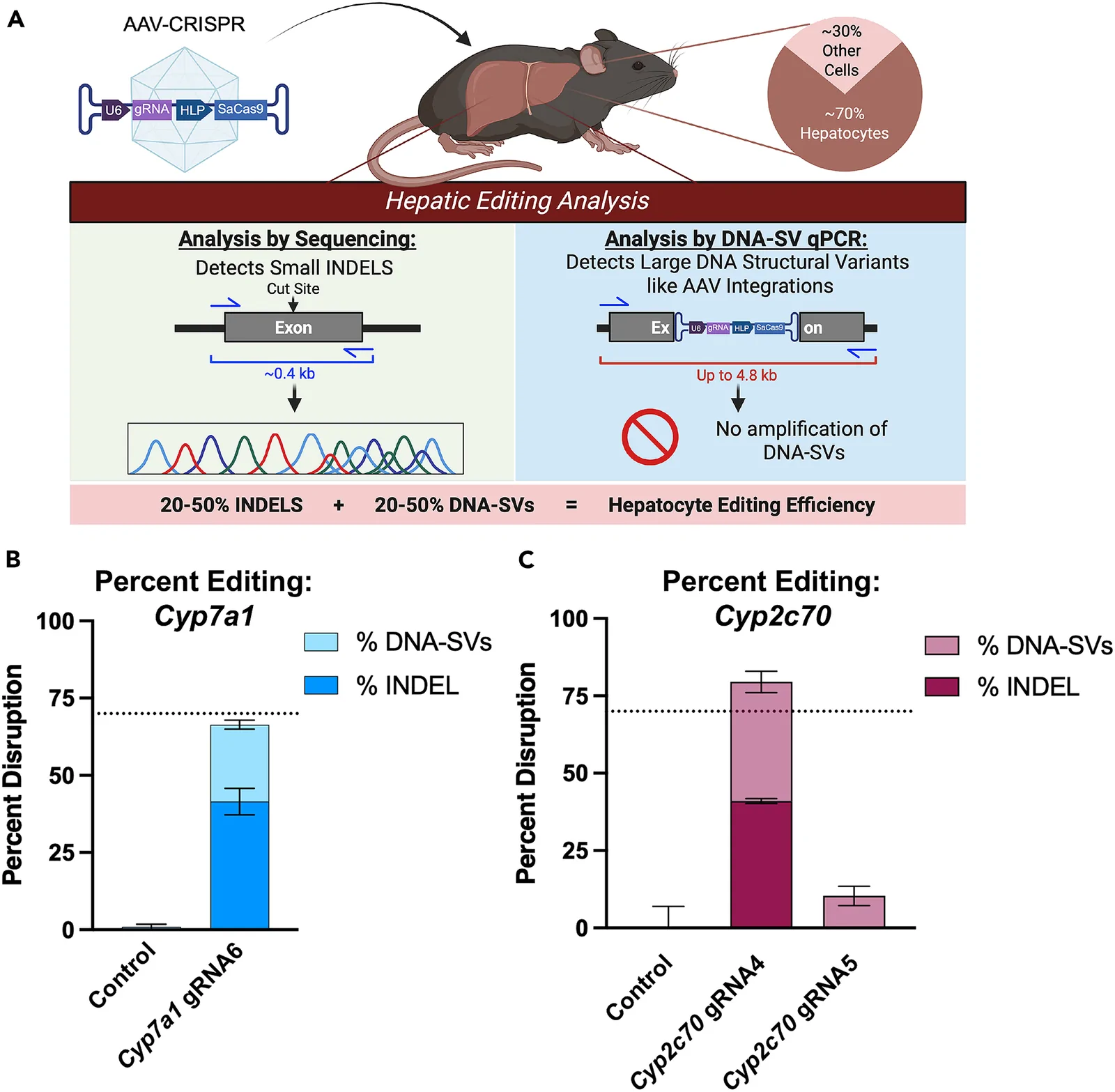
Assessment of hepatic genome editing using INDEL and DNA-SVs (A) Overview of hepatic editing analysis. AAV-CRISPR is primarily delivered to hepatocytes and is limited to hepatocyte-specific expression by the HLP promoter. The liver is approximately 70% hepatocytes, setting the benchmark for liver editing efficiency at approximately 70%. The INDEL percentage is added to the DNA-SV percentage to quantify total editing events. (B) Total INDEL and DNA-SVs in Control mice treated with PBS alone compared to mice injected with an AAV-CRISPR expressing *Cyp7a1* gRNA6. *Cyp7a1* CRISPR approaches 70% editing efficiency. (C) Total INDEL and AAV integration in Control mice treated with PBS alone compared to mice injected with an AAV-CRISPRs expressing gRNAs 4 and 5 targeting *Cyp2c70*. gRNA4, but not gRNA5, reaches 70% editing efficiency and was selected for future analysis. Error bars are standard error of the mean (SEM). n = 4-6 mice/treatment.

***Note:*** While this method accounts for previously unmeasurable editing events, not all editing events will be captured. In addition, since the liver is only 70% hepatocytes, the maximum editing efficiency expected is 70%. Thus, a combined editing efficiency that exceeds 55% is considered efficient disruption, and those gRNAs are normally selected for future studies (Figure 7A).

## Expected outcomes

The use of AAV-CRISPR for hepatic gene disruption can be a powerful tool for biomedical research but quantitative analysis of DNA editing events has not been readily available. Here we present a dual quantification method for different types of editing events, where both INDELs and complex DNA-SV events including AAV integrations are quantified at the target locus. This quantification is especially useful when antibodies are not available for confirmation of protein loss. The example data in Figures 4, 5, 6, and 7 show validation of genome editing for mouse *Cyp7a1*, demonstrating successful editing of the gRNA used to ablate protein expression in our previous work.^1^ Importantly, the data show INDEL levels over 40% (Figure 5) along with DNA-SVs over 20% (Figure 6). Together, nearly 70% disruption was achieved. As the liver is thought to be around 70% hepatocytes,^4^^,^^5^ achieving nearly 70% editing efficiency shows almost complete targeting of hepatocytes in the liver. Finally, we also present data from the optimization of AAV CRISPR targeting *Cyp2c70,* with greater than 70% total editing with gRNA 4 and very little editing with gRNA 5. Based on these results, gRNA 4 was selected for future experiments. This protocol enables researchers to generate and validate AAV-CRISPR tools for efficient hepatic genome editing, thereby streamlining AAV-CRISPR guide RNA design and validation.

## Limitations

There are several limitations to consider in the application of this protocol. First, it was optimized for *Staphylococcus aureus* Cas9 and may require adjustments for other Cas9 orthologs, plasmids, viral delivery systems, or viral dosing. Second, while we have successfully targeted many genes using this approach, some genes may lack desirable gRNA sites. In this case alternate Cas9 orthologs or gene editing strategies should be considered. Third, this protocol is optimized specifically for hepatocytes. Genes expressed primarily in non-hepatocyte cell types will not be targeted using this approach. In addition, delivery of AAV-CRISPR to other organs or tissues will be very different and require optimization for editing quantification. Finally, this approach does not account for off target editing events, though potential off targets are predicted. Therefore, our approach cannot replace deep sequencing and off target editing assessments, which must be performed separately once effective on-target gene disruption has been established.

## Troubleshooting

### Problem 1

There are no suitable gRNAs in the target region or the gRNAs available have high numbers of off targets (See steps 1–3).

### Potential solution

• Increase the search parameters to include up to 10 bp of intronic DNA surrounding the exon. Search again for gRNAs that will still cut within the exon.
• For target regions with few high specificity gRNAs, it is up to the individual user to assess if any of the gRNAs are still usable. When this occurs, targets with exonic off targets can still be selected if the scores indicate it is unlikely a cut will occur. Additional validation may be required for these targets to ensure specificity.

### Problem 2

The AAV ITRs are mutated by restriction digest or whole plasmid sequencing following maxiprep (See step 12).

### Potential solution

• Confirm that NEB Stable competent cells are in use, and ensure that no more than 10 days pass between bacterial transformation and DNA maxiprep.
• Screen more colonies for intact ITRs.
• When replenishing plasmid stocks from a previously generated plasmid, always start with a DNA transformation rather than a glycerol stock. This helps reduce ITR deletions from extended time in bacterial culture.

### Problem 3

AAV genomes are low in the livers of the mice (See the “analysis of AAV genomes in murine liver” section).

### Potential solution

• Review and, if necessary, revisit intraperitoneal injection technique (Step 28) to confirm proper AAV delivery. Both the skin and abdominal layer must be punctured without puncturing intestines. Incorrect technique is the most common reason for low AAV transduction.
• Check viral titers on the AAV preparation by qPCR^19^ to confirm sufficient viral load and reassess the calculations used to dilute virus for injection.
• Confirm no viral infections or other illnesses are occurring in the mouse colony.

### Problem 4

No editing was observed via INDEL or DNA-SVs analyses despite testing multiple gRNAs or the PCRs were inconsistent (See steps 54–76).

### Potential solution

• Confirm gRNA design was completed correctly and that the gRNAs were correctly oriented at cloning. If a G was required for expression from the U6 promoter, verify that it was incorporated.
• For DNA-SVs, especially for amplification from regions with AT-rich regions, further dilution of the DNA and standard curve may be required. Before calling a gRNA a failure, try diluting the DNA and standard curve 1:2 and 1:5 and attempting analysis again.

### Problem 5

DNA editing events cannot be measured (See steps 54–76).

### Potential solution

On rare occasion, the presence of AT-rich or repetitive regions prevents the assessment of editing efficiency at the genomic level. In these cases, we first measure mRNA levels. Most successful gene disruptions also reduce mRNA expression. However, in some rare cases, mRNA expression does not change. When this happens, the mRNA can be assessed for INDELs. This will be most successful for genes which have hepatocyte-specific expression.

• Assess gene expression from hepatic cDNA by standard qPCR methods. If gene expression is not reduced, proceed to measure mRNA INDELs.
• Design primers to amplify the area surrounding the CRISPR gRNA as in Steps 18–24 using the mRNA sequence as the template instead of genomic. Amplicons should be between 350–425 bp with the gRNA site as close to the center of the amplicon as possible.
• Repeat Steps 56–67, substituting diluted cDNA for the genomic DNA in the PCR reaction and using the primers designed for mRNA editing analysis.

***Note:*** In our testing, 500 ng of RNA was converted to cDNA in a final reaction volume of 10 μL. This final reaction was diluted 1:25 in nuclease free water, and 2 μL was used for the PCR reaction.

• Graph the percent INDEL returned from ICE analysis.

In our experience, most AAV-CRISPR editing results in a reduction in mRNA levels. However, disruption of some genes, such as *Cyp7a1*, does not meaningfully reduce mRNA levels. In these instances, we observed nearly 100% of the mRNA transcripts in *Cyp7a1* CRISPR animals were mutated when the region surrounding the gRNA binding site is amplified (Figure 8). In the absence of a CYP7A1 antibody for western blot confirmation, these results could be considered sufficient to warrant future studies.

**Figure 8:**
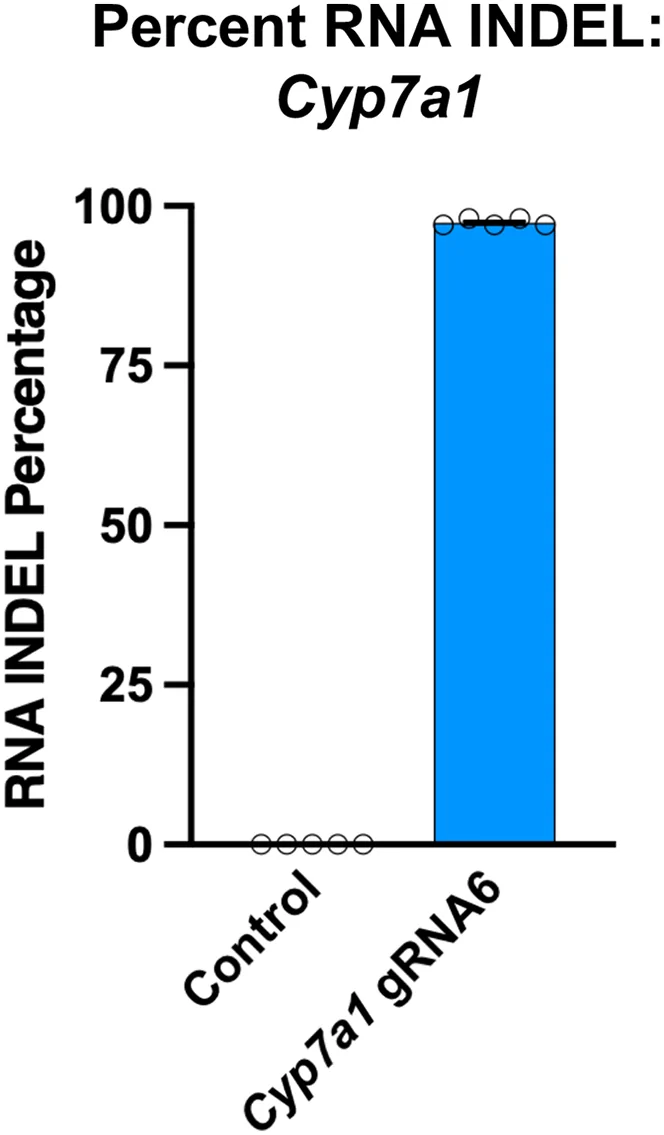
Assessment of INDEL events in mRNA Total INDELs in the mRNA of mice treated with *Cyp7a1* CRISPR approaches 100% editing for this gene with hepatocyte-specific expression. Error bars are standard error of the mean (SEM). n = 4-6 mice/treatment.

## Resource availability

### Lead contact

Requests for further information and resources should be directed to and will be fulfilled by the lead contact, Thomas Q. de Aguiar Vallim (tvallim@mednet.ucla.edu).

### Technical contact

Questions about the technical specifics of performing the protocol should be directed to the technical contact, Kelsey E. Jarrett (kelseyjarrett@g.ucla.edu).

### Materials availability

This study did not generate new unique reagents.

### Data and code availability

This study did not generate code or analyze datasets.

## Acknowledgments

This work is supported by R01DK138340 from the National Institutes of Health to T.Q.d.A.V. T.Q.d.A.V. and E.J.T. are also supported by R01HL174008, R01HL163908, and R01DK128952. E.J.T. is supported by the American Heart Association (23EIA1037961). K.E.J. is supported by the American Heart Association Career Development Award (26CDA1608262), Iris Cantor Women’s Health Center and Clinical and Translational Science Institute pilot grant (UL1TR001881), and UCLA SCORE on Cardiometabolic Health and Disease pilot grant (U54HL170326). K.E.J., and T.Q.d.A.V. are supported by pilot and core voucher grants from the UCLA Clinical and Translational Science Institute (UL1TR001881). R.W.L. is supported by the UCLA Goodman-Luskin Microbiome Center Seed Fellowship and the UCLA Graduate Programs in Bioscience Asrican Sophie & Jack Award.

The lead and senior authors wish to specifically acknowledge the undergraduate research contributions to this work by R.B.-N., C.A.B., P.J.C., R.W.L., O.K.T., S.Y.C., K.S., M.P., and A.S., which were facilitated by research initiatives at UCLA. R.B.-N., P.J.C., K.S., and A.S. participated in research as part of their coursework through the UCLA Biomedical Research Minor. R.B.-N. and C.A.B. were supported by the UCLA Undergraduate Research Fellows Program (URFP). R.B.-N. was supported by the Louis Stokes Alliance for Minority Participation (LSAMP) Fellowship and the Howard Hughes Medical Institute Pathways to Success Program. C.A.B. completed an undergraduate honors thesis through the UCLA Physiological Science program.

The figures were generated using www.Biorender.com.

## Author contributions

K.E.J. conceptualized and designed validation experiments, implemented AAV manufacture, analyzed the data, supervised undergraduate researchers, constructed the protocols, and wrote the manuscript. A.C. and G.E.R. assisted with optimization of AAV production. K.E.J., R.B.-N., C.A.B., P.J.C., R.W.L., O.K.T., S.Y.C., M.P., A.S., R.W.L., K.S., G.E.R., and A.C. tested and validated the gRNA design, primer design, and CRISPR editing protocols. W.R.L. and K.E.J. generated the original gRNA design and *in vivo* AAV quantification protocols from which the protocols shown here were based. W.R.L. edited the manuscript. E.J.T. and T.Q.d.A.V. provided resources, supervision, and contributed to the conceptualization, experimental design, and construction of the protocol and edited the manuscript.

## Declaration of interests

The authors declare no competing interests.

## Declaration of generative AI and AI-assisted technologies in the writing process

To prepare this work, the authors used Claude AI (Anthropic) to reorder steps. The writing in this protocol was completed by the authors, who bear full responsibility for the content.

## Supplemental information

Table S1. AAV Genomes Template, related to Step 52 Template for calculating in vivo AAV genomes from murine liver DNA.

Table S2. DNASV Calculation Example, related to Step 77 Example template for calculating hepatic DNA structural variant (DNA-SV) events.
